# Efficiency of afamin and fibroblast growth factor 21 for early prediction of gestational diabetes mellitus

**DOI:** 10.1038/s41598-026-51453-7

**Published:** 2026-05-11

**Authors:** Abdelrahman O. Sabri, Mostafa M. Elhady, Eman H. A. Hemida, Radwa M. M. Zaki, Amani F. H. Noureldeen

**Affiliations:** 1 Department of Biochemistry, Faculty of Science, Ain Shams University, Cairo, Egypt; 2 Obstetrics and Gynecology Hospital, Ain Shams University, Cairo, Egypt; 3 Obstetrics and Gynecology Department, Faculty of Medicine, Ain Shams University, Cairo, Egypt

**Keywords:** Pregnancy, Gestational diabetes mellitus, Afamin, Fibroblast growth factor 21, ROC curve analysis, Biomarkers, Diseases, Endocrinology, Health care, Medical research, Risk factors

## Abstract

Gestational diabetes mellitus (GDM) is among the most common complications during pregnancy. It is characterized by hyperglycemia that is first identified during pregnancy, typically in the second or third trimesters. However, the relatively late diagnosis limits timely glycemic control and preventive interventions. The present study aimed to evaluate the diagnostic potential of serum afamin and fibroblast growth factor 21 (FGF21) as early predictive biomarkers for GDM. The study involved 17 healthy pregnant women and 26 pregnant women with one or more risk factors for developing GDM. Fasting blood samples were collected in the 1st (8–12 weeks) and 2nd (22–28 weeks) trimesters of pregnancy. Higher serum afamin level was observed in GDM group at 1st trimester (92.9 ± 34.51 ng/ml) compared to normal pregnancy (57.1 ± 25.15 ng/ml). At 2nd trimester, no significant difference in circulated serum afamin was noted between the two groups (73.2 ± 20.30 ng/ml and 68.3 ± 18.74 ng/ml, for GDM and normal pregnancy, respectively). The study also indicated higher serum FGF21 in pregnancy complicated with GDM compared to normal pregnancy at both sampling times (1st trimester: 140.8 ± 32.83 and 97.1 ± 21.43 Pg/ml for GDM and normal; 2nd trimester: 123.8 ± 41.85 and 89.4 ± 32.53 Pg/ml for GDM and normal). At 1st trimester no significant correlations were noted between afamin and all parameters in normal pregnancy, however, in pregnancy complicated with GDM, positive correlations were noted between the hormone and the diabetic profile parameters and baby birth weight. At both 1st and 2nd trimesters, no correlation between FGF21 with all tested parameters, either in normal or complicated pregnancies. ROC curve analysis demonstrated that afamin (AUC = 0.826, *P* < 0.001), FGF21 (AUC = 0.877, *P* < 0.001), and their combined assessment (AUC = 0.9, *P* < 0.001) were strong predictors of GDM development at 1st trimester before the development of the disease than expectant mothers with normal pregnancy.

**Supplementary Information:**

The online version contains supplementary material available at 10.1038/s41598-026-51453-7.

## Introduction

GDM is a worldwide public health concern and is one of the most frequent complications during pregnancy. It relates to multiple adverse pregnancy outcomes, with the prevalence of overweight, obesity and delayed childbearing. It is recognized as one of the most prevalent complications faced during pregnancy^1–4^ and is associated with a marked increase in risks related to both maternal and neonatal health outcomes^4^. Closer monitoring and management before GDM screening later in pregnancy is needed since diagnosis of GDM begins at third trimester which makes it too late for optimal intervention. Previous reports have demonstrated that fetal growth trajectories diverge from 12 weeks onward between pregnancies complicated by GDM and those with normal glucose levels, suggesting that maternal metabolism differences begin at this early stage^5,6^. GDM constitutes a significant health concern, affecting between 15% and 25% of pregnant women globally^7^. In 2018, Egypt was noted as one of the 21 countries with a GDM incidence rate of 15.9%^8–10^. The disease is generally diagnosed through the oral glucose tolerance test conducted between 24 and 28 weeks of gestation^11^. However, this relatively late diagnosis limits timely glycemic control and preventive interventions. Hormonal alterations due to placental secretion make cells less responsive to the effects of insulin which may results in the development of GDM during pregnancy^12^. Monitoring GDM markers in pregnant women can drastically reduce distress, and unnecessary healthcare, and avoid redundant hospitalization. Also, monitoring the disease’s progression enables the optimization of delivery time while minimizing premature births^13^.

Afamin plays a crucial role in antioxidative defense by binding to and transporting vitamin E, an antioxidant key, thereby enhancing the bioavailability and efficacy of vitamin E in mitigating oxidative stress^14^. Multiple pathways connect afamin to insulin resistance in GDM, facilitated by oxidative stress and interactions with key hormones that regulate insulin production. Research indicates that women who later developed GDM exhibited elevated HOMA-IR values, indicative of early insulin resistance, which correlates with heightened afamin levels, especially during the first trimester of pregnancy^15^. Afamin influences insulin signaling pathways through oxidative stress, leading to cellular processes related to oxidative stress that contribute to insulin resistance^16^.

FGF21 is a newly discovered adipokine that is essential for the regulation of glucose and lipid metabolism. This hormone improves insulin sensitivity, lowers triglyceride levels, and maintains energy homeostasis^17^. Significant research has been undertaken on FGF21 analogues as pharmacological agents that promote weight loss, increase energy expenditure, and improve insulin sensitivity in different animal models^18^. The metabolic function of FGF21 has led researchers to hypothesize that this protein may significantly contribute to the pathogenesis of gestational and type 2 diabetes mellitus^19^.

Gestational diabetes mellitus is considered one of the most common complications during pregnancy and it is characterized by hyperglycemia, which is firstly identified during pregnancy typically in the second or third trimesters. Due to our interest in inspecting many diverse cases of complicated pregnancy as well as exploring some potential bioactive markers related to pregnancy^20–24^, the current work was performed and executed. Therefore, we intended and planned to evaluate the diagnostic potential of serum afamin and FGF21, that were both involved in insulin resistance pathways, and the possibility to identify them as biomarkers for early prediction of GDM in expectant women.

## Experimental

The protocol of the current study was approved by Research Ethics committee, Faculty of Medicine, Ain Shams University (FMASU REC). We confirm that all methods reported here were performed in accordance with the relevant guidelines and regulations of the FMASU REC (MS 490/2025), See “Ethics approval and consent to participate” in the Supplementary File. Blood samples were collected from volunteers during their prenatal visits, at 1 st trimester, to the Obstetrics and Gynecology Department, Faculty of Medicine, Ain Shams University. Signed informed consent as well as questionnaire were obtained from each subject before setting the experiments. After a complete explanation of the project, demographic characteristics were recorded in first prenatal visit, including: age, height, weight and blood pressure. Body mass index (BMI) was calculated. Blood pressure of the right arm was taken into a sitting position. The diagnosis of GDM was carried out according to American Diabetes Association, (2019)^25^, depending on the result of an abnormal glucose tolerance test at 24–28 weeks of gestation. GDM was diagnosed using one step approach consisting of an oral glucose overload with 2 h duration that measures plasma glucose concentration.

### Subjects

A total number of forty-three pregnant participants were enrolled in the study. Participants were divided into two main groups:

- Control group: included 17 healthy pregnant women with normal pregnancy without any complications.
- Gestational diabetes mellitus group: included 26 pregnant women with one or more risk factor for GDM development. The risk factors, as indicated by Kouhkan et al. [26a], were: obesity or BMI ≥ 30 Kg/m^2^ (*n* = 16), family history of T2DM (*n* = 9), advanced maternal age ≥ 35 years or older (*n* = 9), prior GDM (*n* = 2). Follow up of the enrolled pregnant women were carried out till delivery of their babies. Time of GDM development was recorded for each patient with recording the type of GDM control. 19 of women with GDM were under diet control, while the rest (*n* = 7) were under insulin treatment. The inclusion criteria for GDM subjects included randomized pregnant women with one or more risk factor for development of GDM at first trimester. At second trimester, after following up, women who developed GDM (follow the practical criteria for diagnosis of GDM) were selected to participate in the study. The exclusion criteria for GDM subjects were hypertension, heart failure, presence of renal or liver disease, active inflammation, intake of medications that may affect glucose metabolism, ischemic heart disease, or pancreatic disease and loss of follow up.

### Methods

- Sample collection: Fasting blood samples were collected from each subject at two gestational periods. One sample was withdrawn at 1 st trimester (8–12 weeks of gestation). The second sample was taken at late 2nd trimester (22–28 weeks gestation). Serum was separated and divided into aliquots, to avoid freezing and thawing, stored at − 80 °C pending assay.
- Biochemical essays: The following parameters were determined using specialized kit for each parameter obtained from different companies:
- Diabetic profile included: fasting blood glucose (FBG), fasting insulin (FI), and glycated hemoglobin (HbA1c). The latter was measured in whole EDTA blood samples. Homeostasis model assessment of insulin resistant (HOMA-IR) was calculated as the following formula developed by Matthews et al^26^..
- Liver function parameters including serum albumin, activities of both alanine amino transferase (ALT) and aspartate amino transferase (AST).
- Kidney function parameters including serum urea, creatinine and uric acid.
- Serum afamin using ELISA kit obtained from Bioassay Technology Laboratory (BT LAB), Zehjiang, China (Cat. no. E3471Hu). The detection range was 5–600 ng/ml. The kit’s sensitivity was 2.83 ng/ml.
- Serum FGF1 using ELISA kit obtained from Bioassay Technology Laboratory (BT LAB), Zehjiang, China (Cat. no. E1983Hu). The detection range was 7–1500 Pg/ml. The kit’s sensitivity was 3.52 Pg/ml.

Laboratory analyzer used for the measurement of afamin and FGF21 was Stat Fax-2100 absorbance microplate readers. Laboratory analyzers used for the measurement of the rest of biochemical parameters was a UV/VIS spectrophotometer model T90+.

### Statistical analysis

Data was entered and evaluated using Social Science Statistical Software (SPSS V.23). The suitability of the quantitative data for normal distribution was tested by the Shapiro–Wilk test. Descriptive statistics (median, ± standard deviation, mean, interquartile range) were performed to examine the distribution of explained data. The student’s t test was used for the comparison of two groups of quantitative data showing normal distribution, and the Mann–Whitney U test was used for the comparison of two groups of data with abnormal distribution. The Pearson chi-square test was used to compare qualitative data. One-way ANOVA (analysis of variance) test was used for the comparison of three groups of quantitative data showing normal distribution. Pearson’s correlation test was used to get correlation coefficient (*r*) and a significance (*p*) that indicate strength, direction, and statistical significance of the relationship between variables. Receiver operating characteristic (ROC) analysis was used to test the ability of maternal serum afamin and FGF21 concentrations to discriminate between pregnant women who developing GDM and those that do not. *P* < 0.05 was considered statistically significant.

## Results

### Initial characteristics and delivery outcomes of normal pregnant women and GDM groups

Characteristics and delivery outcomes obtained from hospital records of the recruited pregnant women are indicated in Table 1. Significant differences were found between normal pregnant women and GDM groups in terms of maternal age, BMI and blood pressure (both systolic and diastolic). Data also indicated that pregnant women with GDM had delivered their babies earlier and heavier than babies delivered to normal pregnant women. Furthermore, 80% of women with GDM had caesarian section compared to 25% women with normal pregnancy.

**Table 1:** Initial characteristics and delivery outcomes of normal pregnant women and GDM risk groups.

Groups parameters	Normal (*n* = 17)	GDM (*n* = 26)	*P*
Maternal age (years)	24.4 ± 3.27	33.3 ± 5.73	0.001
Gestational age at 1 st trimester (weeks)	10.0 (1.4)	10.5 (3.0)	0.195 (NS)
Gestational age at 2nd trimester (weeks)	25.3 ± 1.61	26.0 (3.2)	0.122 (NS)
BMI at sampling time (kg/m^2^)	23.1 (2.1)	27.8 (2.3)	0.001
Systolic blood pressure at 1 st trimester (mmHg)	65.3 ± 9.91	75.8 ± 8.16	0.003
Diastolic blood pressure at 1 st trimester (mmHg)	107.5 ± 13.00	117.2 ± 12.17	0.039
Delivery outcomes a- Birth weight (Kg) b- Mode of Delivery: 1. Vaginal Delivery 2. Cesarean Delivery c- Gestational Age at Delivery (weeks)	2.8 ± 0.26 75% 25% 38.7 ± 1.38	3.3 ± 0.52 20% 80% 35.0 ± 2.09	0.008 0.034 0.001

Data are presented as mean ± standard deviation, median (Inter-quartile range) or frequency (%). BMI: body mass index.

*P* value ≤ 0.05 is significant, *P* value > 0.05 is non-significant, NS: non-significant.

### Diabetic profile, liver and kidney function parameters for normal pregnant women and GDM groups

As indicated in Tables 2 and 3, diabetic profile parameters (FBG, fasting insulin, HbA1c and HOMA-IR) were significantly elevated in risk/or GDM groups compared to the matched control group, both at 1 st and at 2nd trimesters. Regarding liver function parameters, results revealed significant elevation in ALT median value in pregnant women with GDM relative to its matched value in normal pregnancy at 1 st trimester, while other parameters showed no significant difference between groups. Furthermore, data at 2nd trimester indicated no significant differences in liver function parameters between normal pregnancy and pregnancy complicated with GDM. Additionally, kidney function, as indicated by measuring, serum urea and uric acids, showed significant increase in GDM group compared to normal pregnant group both at 1 st and 2nd trimesters.

**Table 2:** Diabetic profile, liver and kidney function parameters of normal control and risk groups at 1 st trimester.

Groups parameters	Normal *n* = 17	Risk *n* = 26	*P*
FBG (mmol/L) % change	4.5 ± 0.33	5.1 ± 0.52 11.07	0.01
FI (mIU/ml) % change	9.6 ± 1.12	13.0 ± 2.39 34.02	0.001
HbA1c (%) % change	4.9 ± 0.32	5.4 ± 0.29 10.83	0.001
HOMA-IR % change	1.9 ± 0.34	2.6 ± 0.53 36.26	0.001
ALT (U/L) % change	10.0 (4.5)	13.0 (6.0) 30	0.004
AST(U/L) % change	17.5 ± 7.6	15.1 ± 6.2 11.11	0.263 (NS)
Albumin (g/L) % change	42.0 ± 4.50	42.0 ± 7.20 2.38	0.896 (NS)
Urea (mmol/L) % change	2.9 ± 0.58	3.3 ± 0.80 16.97	0.045
Creatinine (µmol/L) % change	61.8 ± 10.60	61.8 ± 9.72 4.34	0.437 (NS)
Uric acid (µmol/L) % change	172.5 ± 31.53	202.3 ± 5.95 11.11	0.026

Data are presented as mean ± standard deviation. FBG: Fasting blood glucose, FI: Fasting insulin, HbA1c: glycated Hemoglobin, HOMA-IR: Homeostatic Model Assessment of Insulin Resistance, ALT: Aspartate Aminotransferase, AST: Aspartate Aminotransferase.

*P* value ≤ 0.05 is significant. *P* value > 0.05 is non-significant, NS: non-significant.

**Table 3:** Diabetic profile, liver and kidney function parameters of normal control and GDM groups at 2^nd^ trimester.

Groups parameters	Normal *n* = 17	GDM *n* = 26	*P*
FBG (mmol/L) % change	4.5 ± 0.32	5.4 ± 0.73 16.03	0.001
FI (mIU/ml) % change	8.6 ± 1.33	13.5 ± 3.00 53.40	0.001
HbA1c (%) % change	4.8 ± 0.22	5.9 ± 0.54 23.02	0.001
HOMA-IR % change	1.8 ± 0.28	3.3 ± 1.00 69.49	0.001
ALT (U/L) % change	11.2 ± 3.20	14.6 ± 6.83 25.33	0.063 (NS)
AST(U/L) % change	14.6 ± 4.04	13 ± 4.63 10.83	0.256 (NS)
Albumin (g/L) % change	42.0 ± 8.20	41.0 ± 5.90 0.48	0.477 (NS)
Urea (mmol/L) % change	2.7 ± 0.50	3.0 ± 0.46 13.14	0.023
Creatinine (µmol/L) % change	53.04 ± 6.19	53.04 ± 6.19 4.76	0.221 (NS)
Uric acid (µmol/L) % change	166.6 ± 54.74	220.1 ± 59.5 30.24	0.01

### Serum afamin and FGF21 in normal and GDM groups

**Fig. 1:**
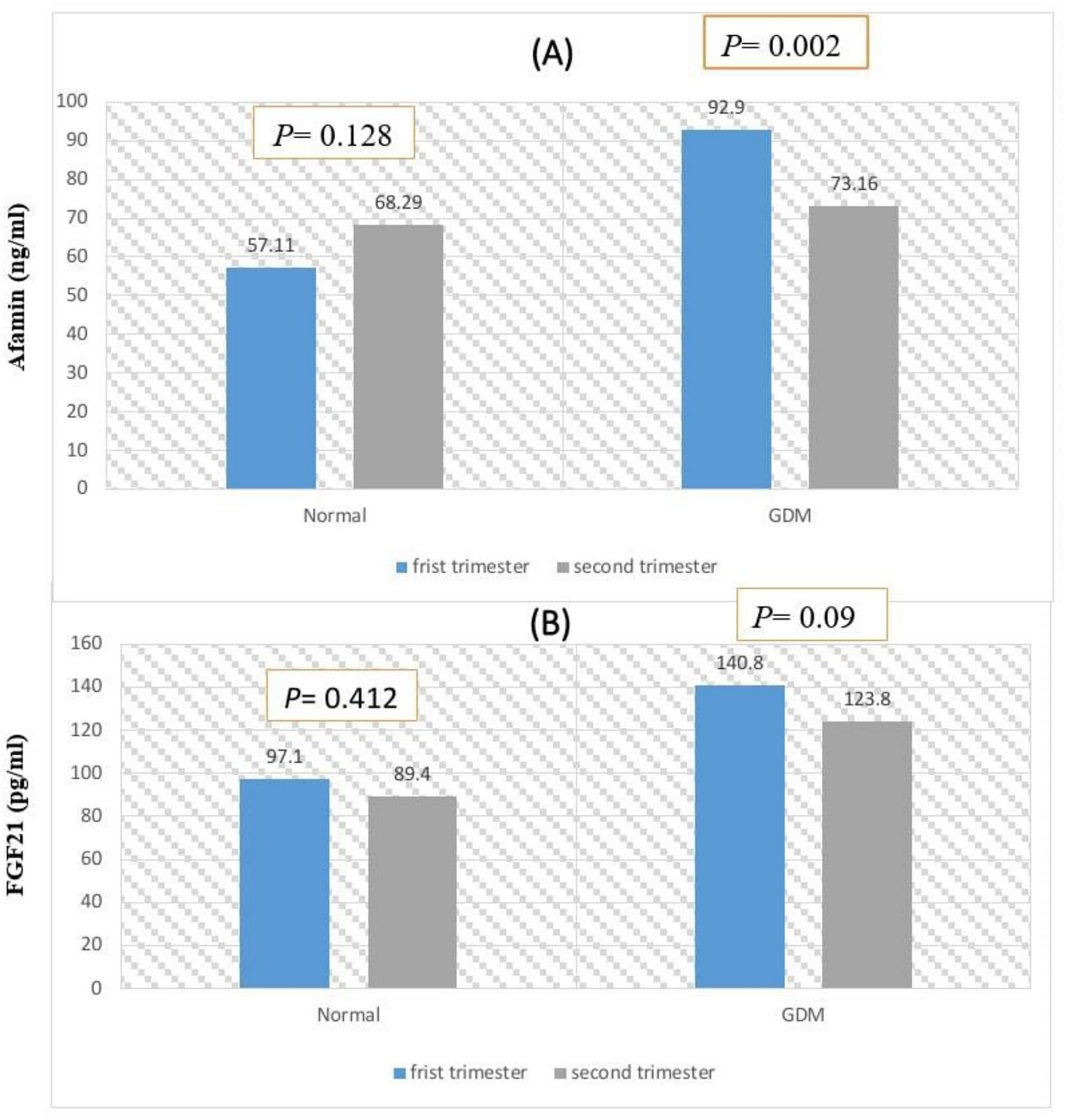
**(A)** Difference in circulated serum afamin between 1 st and 2nd trimesters in normal pregnancy and in GDM groups; **(B)** Difference in circulated serum FGF-21 between 1 st and 2nd trimesters in normal pregnancy and in GDM groups.

As shown in Table 4, serum afamin was significantly elevated in complicated pregnancy compared to normal pregnancy at 1 st trimester. It is interesting to point out to the matched serum afamin between normal and GDM groups at 2nd trimester. Results also revealed significantly decreased serum afamin in pregnancy complicated by GDM at 2nd trimester compared to 1 st trimester, although it was not the case in healthy pregnancy, where lack of significance was noted in serum afamin mean values between 1 st and 2nd trimesters (Fig. 1A). On the other hand, data indicated significant elevation in serum FGF21 in complicated pregnancy both at 1 st and 2nd trimesters compared to their matched values in normal pregnancy (Table 4). No significant change in circulated FGF21 in GDM between 1 st and 2nd trimesters. Same results were noted for normoglycemic pregnancy (Fig. 1B).

**Table 4:** Serum afamin and FGF21in normal control and GDM groups at 1^st^ and 2^nd^ trimesters.

Groups parameters	1 st trimester		*P*	2nd trimester		*P*
	Normal *n* = 17	Risk *n* = 26		Normal *n* = 17	GDM *n* = 26	
Serum Afamin (ng/ml) % change Range	57.1 ± 25.15 (12–96)	92.9 ± 34.51 57.6 (52–228)	0.001	68.3 ± 18.74 (37–103)	73.2 ± 20.30 7.1 (37–107)	0.43 (NS)
Serum FGF21 (Pg/ml) % change Range	97.1 ± 21.43 (53–149)	140.8 ± 32.83 (92–221)	0.001	89.4 ± 32.53 (51–171)	123.8 ± 41.85 21.36 (56–227)	0.006

P value ≤ 0.05 is significant, P value > 0.05 is non-significant, NS: non-significant.

### Correlation study

- Correlations between afamin and studied parameters: At 1 st trimester, no significant correlations were noted between afamin and all parameters in normal pregnancy. However, in pregnancy complicated with GDM, positive correlations were noted between afamin and each of: BMI, FBG, FI, HbA1c, HOMA-IR, ALT and baby birth weight (Table S1). At 2nd trimester, in normal control, afamin was correlated with FI, while in GDM it was correlated with FBG (Table S2).
- Correlations between FGF21 and parameters studied: Both at 1 st and 2nd trimesters, almost absence of significant correlations between the hormone and all tested parameters either in normal or complicated pregnancies (Table S3 and Table S4).

### The diagnostic accuracy of afamin at 1 st and 2nd trimesters

**Fig. 2:**
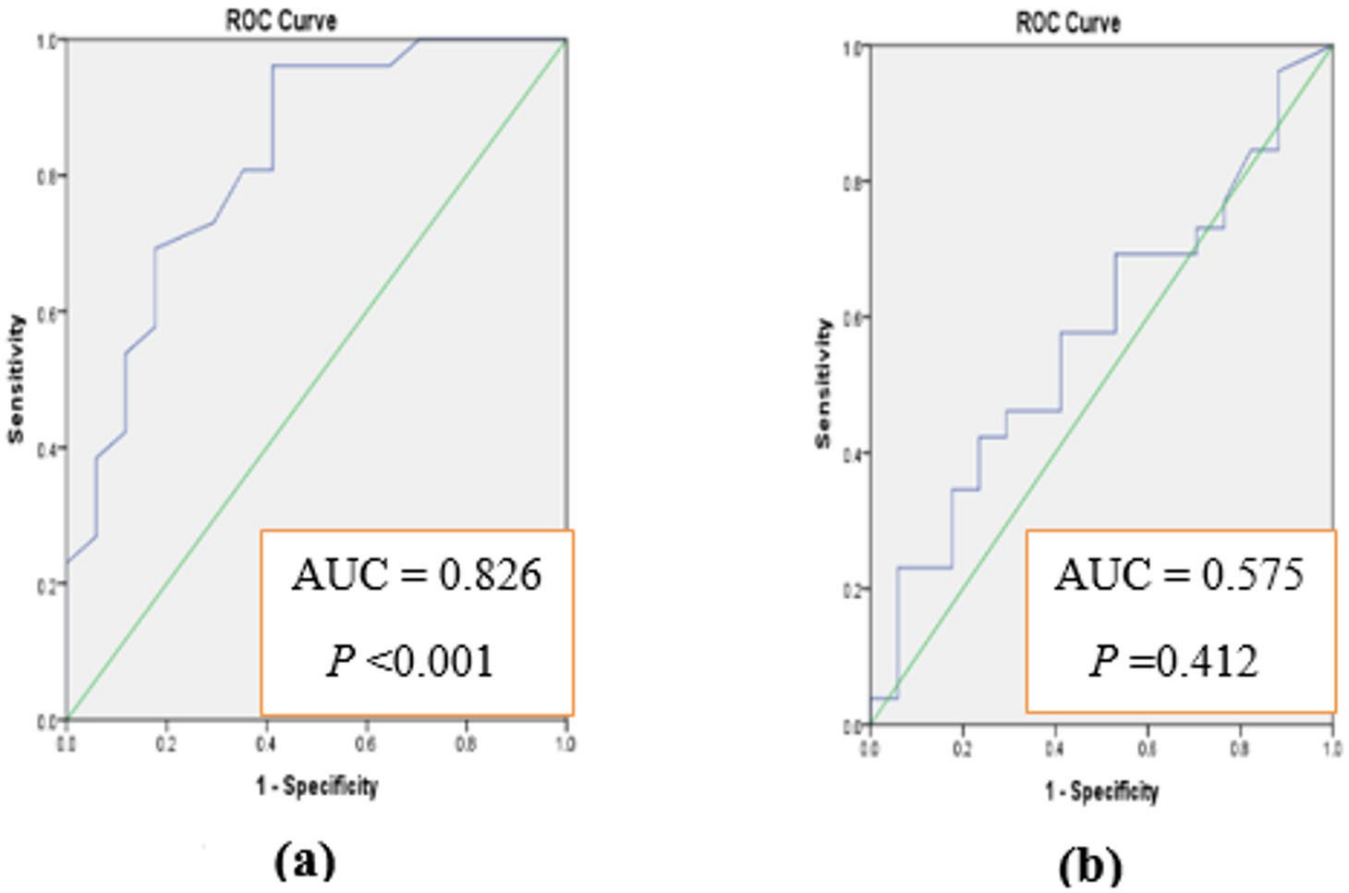
ROC curve analysis using afamin for discriminating GDM from normal pregnancy at 1 st (**a**) and at 2nd (**b**) trimesters.

**Fig. 3:**
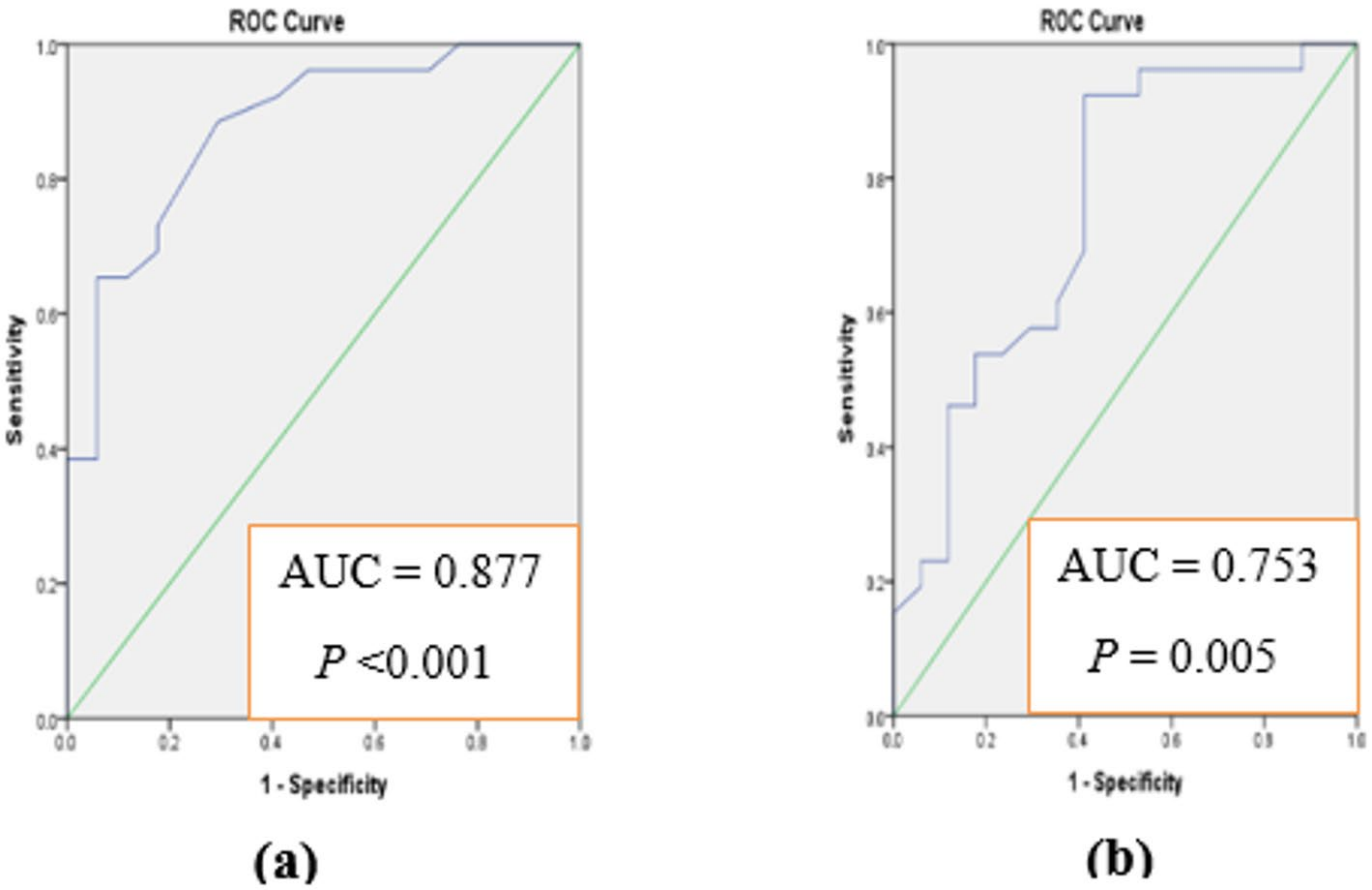
ROC curve analysis using FGF21 for discriminating GDM from normal pregnancy at 1 st (**a**) and 2nd (**b**) trimesters.

**Fig. 4:**
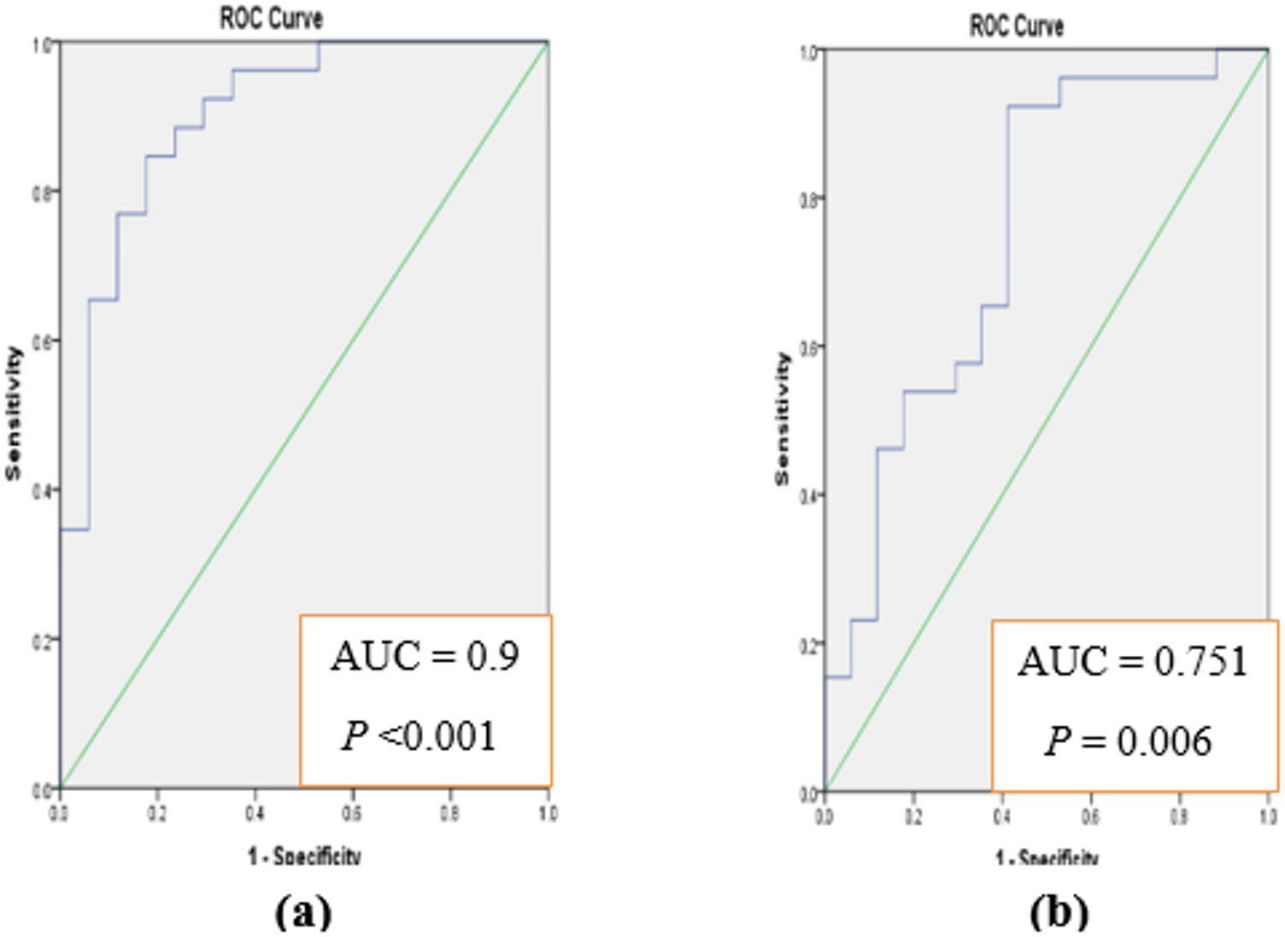
ROC curve analysis using merged afamin and FGF21 for discriminating GDM pregnancy from normal pregnancy at 1 st (**a**) and 2nd (**b**) trimesters.

It was of our interest to analyze the diagnostic accuracy of both markers for predicting GDM early before developed. Receiver Operating Characteristic (ROC) curves were constructed and area under each curve was calculated to evaluate the sensitivity and specificity. Results presented in Fig. 2a indicate that serum afamin could be efficient for early prediction of GDM at 1 st trimester. Furthermore, ROC curves (Fig. 3a and Table S5) indicate the ability of FGF21 to differentiate between normal pregnancy and pregnancy progressed to GDM at 1 st trimesters. Since both serum afamin and serum FGF21 showed promising variations between pregnant women who developed GDM and those with normal pregnancy at 1 st trimester, we were interested to analyze the diagnostic accuracy of merged afamin and FGF21 for predicting GDM early before developed. Figure 4a and Table S5 indicates the ability of merged afamin and FGF21 to early differentiate between normal pregnancy and pregnancy progressed to GDM later during pregnancy at 1 st trimester.

## Discussion

The current study examined two biochemical markers, afamin and FGF21, for early prediction of GDM. The study included two groups of expecting women: normotensive pregnant women and pregnant women group with suspected GDM. The latter group included pregnant women with one or more risk factor for developing GDM. Blood samples were withdrawn at 1 st trimester (8–12 weeks) and before the development of GDM. The second sample was taken at 2nd trimester (22–28 weeks). Both normal control group and GDM were matched for gestational age. However as expected, compared to control group, GDM group was older and obese. Data, at both trimesters, indicated elevated diabetic profile parameters (FBG, fasting insulin, HbA1c and HOMA-IR) in GDM group compared to normal pregnant group, indicating increased insulin resistance.

Afamin is important in antioxidative defense because it binds and transports to vitamin E, making vitamin E more bioavailable and potent for combating oxidative stress^27^. The relationship between afamin and pregnancy related complications has been investigated in few studies. In our study, circulated serum afamin mean value was statistically higher in pregnant women who later developed GDM compared to normal pregnancy at 1 st trimester, however, it did not reach statistical significance between the two studied groups at 2nd trimester. There is recent evidence of significant difference between afamin levels at 1 st trimester being elevated in GDM patient group compared to normal pregnancy^28^. Moreover, significantly higher afamin concentration in serum was indicated for women, who later developed GDM, at 1 st trimester compared to normal control pregnancy^29^. Furthermore, it was previously reported that afamin levels in pregnancies affected by GDM in the 2nd trimester do not significantly differ from healthy pregnancies^27,30^. It was also concluded that increased afamin was an important predictor for GDM development^31^. Higher serum afamin in GDM patients than normal pregnant women group at 1 st trimester was reported, although it did not reach statistical significance^32^. In addition, it was recently indicated that afamin levels could serve as a potential biomarker for early prediction of GDM^33^. The authors reported similar results to ours with increased afamin in 1 st trimester, although in contrast to our data, they noticed elevated serum afamin in the 2nd trimester in GDM group.

In this study, GDM group was presented with significantly decreased serum levels of afamin in the 2nd trimester compared to its matched value in the same group at 1 st trimester. This could be due to enhanced oxidative stress as GDM developed, which may result in increased utilization of afamin as an antioxidative defense mechanism^34^. Moreover, our result indicated elevated serum afamin in normal pregnancy at 2nd trimester compared to 1 st trimester, but without significant difference. Some reports indicated that in pregnancies complicated by GDM, afamin exhibited a markedly decreased lag time for detection of GDM in pregnancy, suggesting its use as an early biomarker to assess risk for GDM development during the early weeks of gestation^28,32^. During healthy pregnancy, reports had indicated gradually increased afamin levels, beginning in the 1 st trimester, and throughout gestation^27,35^. This probably could be due to altering hormonal levels and consequent regulation of the afamin gene expression in the human liver by the hormones. The increase in afamin is essential to counteract the rise in oxidative stress associated with pregnancy and indicates the role of afamin in ensuring that the body successfully adapts to the increasing metabolic demands. Vascular tendencies and appropriate uterine tone for a healthy pregnancy require reactive oxygen species and reactive nitrogen species pathway, which are modified by afamin and regulate oxidative equilibrium^36^. In addition, its antioxidant properties protect cells against the harmful effects of high oxidative stress and prevent the development of preeclampsia^37,38,39^.

Our obtained data from hospital records indicated that pregnant women with GDM had delivered their babies earlier and heavier than those delivered to normal pregnant women. 20% of GDM patients gave their babies through vaginal mode compared to 75% of cases with normal pregnancy. Additionally, we obtained a positive correlation between increased serum afamin and baby birth weight in GDM but not in normal pregnancy, which may indicate an association between afamin and increased fetal growth. Previous reports also declared that increased afamin in GDM mothers with large for gestational age (LGA) fetuses than appropriate for gestational age (AGA) fetuses^27,40^. In addition, afamin could improve pregnancy outcomes by its interactions with cytokines like TNF-α and probably through modulations of transcription factors like NF-κB and PPARγ that are involved in the control of inflammation and oxidative stress^41,42^. Furthermore, it was pointed out that the increased afamin expression in umbilical cord blood of neonates of diabetic mothers, independent of other clinical parameters underscoring the role of afamin in fetal outcome^43^.

The sensitivity and specificity of afamin in the prediction of GDM has been evaluated in our study. Data from the constructed ROC curve pointed out to the reasonable value of using afamin as a promising biomarker for GDM at 1 st (AUC = 0.826, *P* ≤ 0.001, sensitivity = 96.2% & specificity = 58.82%) but not at 2nd (AUC = 0.575, *P* = 0.412, sensitivity = 42.3% & specificity = 70.58%) trimester. Previous data also indicated the sensitivity and specificity of afamin in prediction of GDM were 69.2% and 68.3%, respectively^44^.

FGF21, an adipokine, secreted from adipose tissue, is known to regulate glucose and lipid metabolism. It enhances insulin sensitivity, lowers triglycerides and regulates energy homeostasis^21^. The metabolic role of FGF21 let the researchers suggest that this hormone can play an important role in the pathogenesis of GDM and T2DM^19^. Few studies had compared levels of circulating FGF21 GDM and normoglycemic pregnancy at 1 st trimester, although the majority were carried out at late second and at third trimesters after GDM had developed. In this study, the changes in circulating serum FGF21 in normal pregnancy and in pregnancy complicated with GDM by advancing gestation (both at 1 st and 2nd trimesters) were assessed in order to evaluate the association between the hormone and GDM before recommended routine screening for the latter.

According to the obtained results, and compared to normal pregnant group, serum FGF21 levels were significantly higher in GDM patients, at both trimesters, before and after GDM had developed. Previous investigations had also indicated increased early pregnancy levels of FGF21 in GDM at gestational age 6–15 weeks^45^. Notably, the authors observed this association between FGF21 and GDM primarily in individuals with overweight or obesity (BMI ≥ 24 kg/m^2^). However, FGF21 increased significantly at 14–21 gestational weeks before the routine GDM diagnosis time, regardless of whether analyzed overall or by corresponding BMI subgroup^46^. It would be ascribed to report that most studies indicated increased FGF21 in GDM after 24 weeks gestation. At late 2nd trimester, it was noted significantly higher levels of the hormone in GDM group than their matched value in normal pregnancy^47,48^. Another study revealed that levels of FGF21 were higher in the GDM group compared to the normal glucose tolerance group at gestational age 24–28 weeks^49^. At 2nd trimester, higher serum FGF21 in GDM pregnant women compared to control was also reported by different researchers^50–52^. Elevated FGF21 in GDM was found compared to normal pregnancy^48^. It is worth mentioning that there are some contradictions and inconsistences in the available literature regarding circulated FGF21 levels in GDM and normal pregnancy. Although a group of researchers indicated comparable levels of the hormone between GDM and normal pregnancy, other groups indicated decreased levels. For example, some studies reported non-significant differences in FGF21 levels between the GDM group and normal control pregnant women^53–56^. Furthermore, comparable circulating levels of FGF21 between GDM and normal control were also reported^54,57^. On the other hand, down-regulation of FGF21 in GDM was also indicated^58^. All the above-mentioned studies indicating unchanged or decreased FGF21 in GDM, were not consistent with its effect on glucose metabolism reported in animal studies^59,60^.

The present investigation suggested that the increased FGF21 levels in patients with T2DM and GDM might result from the body’s compensatory mechanism similar to hyperinsulinemia, to improve the pathological state. As the expression of FGF21 receptor 1 and β-klotho receptor in white adipocytes decreased, FGF21 cannot bind properly to form co-receptors, resulting in the compensatory increase in circulating FGF21 levels. On the other hand, due to metabolic disorders and reduced sensitivity to FGF21, the liver must compensatory synthesize and secrete more FGF21 to maintain metabolic homeostasis^61,62^. Furthermore, the ability of FGF21 to promote insulin secretion was previously explained as enhancing the expression of insulin gene transcription factors and N-ethylmaleimide-sensitive factor attachment protein receptor protein, thereby activating the phosphatidylinositol 3-kinase/Akt signaling pathway^63^.

In the current work, GDM women had higher BMI values compared to normal pregnant women (16 of the enrolled GDM women were obese with BMI ≥ 30 kg/m^2^). We were not able to obtain significant association between FGF21 with BMI in control pregnant group or in GDM subjects either under diet control or insulin treatment. This finding might roll out the relation between this protein and BMI. It was previously reported significant differences in FGF21 levels at 14-21weeks gestation between women with or without GDM, irrespective of BMI status^44^. It was also indicated that these differences were more pronounced among women with a BMI ≥ 28 kg/m^2^ compared to those with a BMI < 24 kg/m^2^^44^. Furthermore, it was speculated by other study^64^ that elevated FGF21 levels might not directly contribute to insulin resistance but could be secondary to the effects of obesity on insulin and FGF21 sensitivity.

In the current work, the relationship between FGF21 and the selected biochemical parameters were assessed. Although we noticed significantly increased levels of diabetic markers (reflecting increased insulin resistance) in GDM compared to non GDM groups, however, we did not find significant correlations between FGF21 and any of these markers. Our results indicated absence of significant correlations between the hormone with markers of liver and kidney functions or baby birth weight. Consistent with our findings, several researchers reported no significant correlation between FGF21with fasting insulin and HOMA-IR^55,65,66^. These findings could be returned to the predominance of low HOMA-IR values among the tested participants^55^. However, these results were not consistent with our GDM participants who were presented with elevated diabetic markers. On the other hand, previous studies had found contradicted data as they indicated a positive correlation between FGF21 with insulin resistance in women with GDM^44,52^.

The data of ROC curve analysis of FGF21 concluded that the hormone could be valuable in predicting GDM at early pregnancy. At 1 st trimester, the values of AUC, sensitivity and specificity were: 0.877, 65.38% and 88.23%, respectively, compared to their matched values at 2nd trimester (0.753, 92.30% and 58.52% for AUC, sensitivity and specificity, respectively).

As both serum afamin and FGF21 showed promising variations at early pregnancy and before the development of GDM, the diagnostic accuracy of merged afamin and FGF21 were analyzed. The obtained data from the constructed ROC curve indicated the ability of both markers to discriminate pregnancy with risk for GDM at 1 st trimester that will be progressed to GDM later during 2nd trimester. The data obtained from the analysis at 1 st sampling were: AUC = 0.9 with sensitivity and specificity equivalent to 91.66% and 78.94%, respectively. The corresponding values at 2nd trimester were 0.75, 77.41% and 83.3% for AUC, sensitivity and specificity, respectively. These demonstrated data, which combine both afamin and FGF21 together may be promising and efficient biomarkers to early predict GDM symptoms.

The current study concluded that both afamin and FGF21 were considered efficient biomarkers for predicting GDM early before developed. Although the small total sample (*n* = 43) and the imbalance between the experimental and control groups (*n* = 17) were limitations that might affect the statistical power, our results did find significant differences, which indicated that the study was adequately powered to detect the observed effects, even if the power was theoretically low. The single-center design and limited sample size may limit the generalizability of our findings to the broader pregnant population. However, the results should be considered as a preliminary study, and future multicenter studies with larger sample sizes are needed to confirm our findings and establish their external validity. Thereby necessitating caution when applying these results to different clinical settings, geographical, socioeconomic, and institutional factors unique to our center may have influenced the outcomes.

## Conclusion

Our study showed that both afamin and FGF21 were higher in pregnant women with risk for developing GDM at 1 st trimester and before the development of the disease than expectant mothers with normal pregnancy. Beyond the small sample size, statistical power was able to detect subtle difference between GDM and matched control regarding the two examined proteins. Therefore, the current study concluded that combination of both afamin and FGF21 may be promising and efficient biomarkers to early predict GDM symptoms. However, additional research is required to validate the conclusion and to further examine the role of afamin and FGF21 in the development and progression of GDM.

## Supplementary Information

Below is the link to the electronic supplementary material.

Supplementary Material 1

Supplementary Material 2

## Data Availability

Any datasets used that support the findings of this study are available from the corresponding author upon reasonable request. A supplementary file, containing the table of correlations study between afamin and FGF21 with biochemical parameters and clinical characteristics at 1 st trimester and 2 nd trimester, diagnostic values of afamin, FGF21 and merged afamin with FGF21 at 1 st and 2 nd trimesters in normal pregnancy and GDM groups.

## References

[1] Reitzle, L. et al. Gestational diabetes in Germany: Development of screening participation and prevalence. *J. Health Monit.* **6**, 3–18 (2021). 35146306 10.25646/8325 PMC8734204

[2] Venkatesh, K. & Landon, M. Diagnosis and management of gestational diabetes. *Contemp. OB/GYN.* **66**, 9–15 (2021).

[3] McIntyre, H. D. et al. Gestational diabetes mellitus. *Nat. Rev. Dis. Primers* **5**, 47–65 (2019). 31296866 10.1038/s41572-019-0098-8

[4] Ferrocino, I., Ponzo, V., Gambino, R. & Zarovska, A. Changes in the gut microbiota composition during pregnancy in patients with gestational diabetes mellitus (GDM). *Sci. Rep.* **8**, 12216–12228 (2018). 30111822 10.1038/s41598-018-30735-9 PMC6093919

[5] Brand, J. S. et al. Gestational diabetes and ultrasound-assessed fetal growth in South Asian and White European women: findings from a prospective pregnancy cohort. *BMC Med.* **16**, 203–215 (2018). 30396349 10.1186/s12916-018-1191-7 PMC6219043

[6] Sovio, U., Murphy, H. R. & Smith, G. C. Accelerated fetal growth prior to diagnosis of gestational diabetes mellitus: A prospective cohort study of nulliparous women. *Diabetes Care* **39**, 982–987 (2016). 27208333 10.2337/dc16-0160

[7] Mazumder, T., Akter, E., Rahman, S. M., Islam, M. T. & Talukder, M. R. Prevalence and risk factors of gestational diabetes mellitus in Bangladesh: Findings from demographic health survey 2017–2018. *Int. J. Environ. Res. Public. Health* **19**, 2583–2592 (2022). 35270274 10.3390/ijerph19052583 PMC8909680

[8] Wang, H. et al. IDF diabetes atlas: Estimation of global and regional gestational diabetes mellitus prevalence for 2021 by International Association of Diabetes in Pregnancy Study Group’s Criteria. *Diabetes Res. Clin. Pract.* **183**, 109050–109056 (2022). 34883186 10.1016/j.diabres.2021.109050

[9] Eltoony, L. F., Ibrahem, S. A., Hafez, M. Z., Ali, O. M. & Elsewify, W. A. Prevalence and risk factors for gestational diabetes in Aswan, Egypt according to International Association of the Diabetes and Pregnancy Study Groups (IADPSG). *Egypt. J. Hosp. Med.* **82**, 701–707 (2021).

[10] Muche, A. A., Olayemi, O. O. & Gete, Y. K. Prevalence and determinants of gestational diabetes mellitus in Africa based on the updated international diagnostic criteria: A systematic review and meta-analysis. *Arch. Public Health* **77**, 36–55 (2019). 31402976 10.1186/s13690-019-0362-0 PMC6683510

[11] Hu, J. et al. Association of maternal lipid profile and gestational diabetes mellitus: A systematic review and meta-analysis of 292 studies and 97,880 women. *E Clinical Medicine* **34**, 100832–100838 (2021). 10.1016/j.eclinm.2021.100830 PMC8102708 33997732

[12] Padhi, S., Nayak, A. K. & Behera, A. Type II diabetes mellitus: A review on recent drug based therapeutics. *Biomed. Pharmacother* **131**, 110708.-110730. (2020). 32927252 10.1016/j.biopha.2020.110708

[13] Belovic, D. K., Plešinac, S., Dotlić, J. & Radojević, A. S. Biochemical markers for prediction of hypertensive disorders of pregnancy. *J. Med. Biochem.* **38**, 71–82 (2019). 30820186 10.2478/jomb-2018-0001 PMC6298456

[14] Hubalek, M. et al. The vitamin E-binding protein afamin increases in maternal serum during pregnancy. *Clin. Chim. Acta* **434**, 41–47 (2014). 24768783 10.1016/j.cca.2014.03.036 PMC4065568

[15] Köninger, A., Iannaccone, A., Hajder, E., Frank, M. & Schmidt, B. Afamin predicts gestational diabetes in polycystic ovary syndrome patients preconceptionally. *Endocr. Connect.* **8**, 616–624 (2019). 30991357 10.1530/EC-19-0064 PMC6510713

[16] Juhász, I. et al. Afamin levels and their correlation with oxidative and lipid parameters in non-diabetic, obese patients. *Biomolecules* **12**, 116–126 (2022). 35053264 10.3390/biom12010116 PMC8773538

[17] Velingkar, A., Vuree, S., Prabhakar, P. K. & Kalashikam, R. R. Fibroblast growth factor 21 as a potential master regulator in metabolic disorders. *Am. J. Physiol. Endocrinol. Metab.* **324**, E409–E424 (2023). 36629821 10.1152/ajpendo.00244.2022

[18] Yan, J. et al. The roles and pharmacological effects of FGF21 in preventing aging-associated metabolic diseases. *Front. Cardiovasc. Med.* **8**, 655575–655585 (2021). 33869312 10.3389/fcvm.2021.655575 PMC8044345

[19] Yuan, D., Wu, B. J., Henry, A., Rye, K. A. & Ong, K. L. Role of fibroblast growth factor 21 in gestational diabetes mellitus: A mini-review. *Clin. Endocrinol. (Oxf)* **90**, 47–55 (2019). 30346647 10.1111/cen.13881

[20] Alshannag, F., Zaki, R. M. M., Hemida, E., ElBakry, M. M. M. & Noureldeen, A. F. H. Endostatin and cystatin C as potential biomarkers for early prediction of preeclampsia. *ACS Omega*. **8**, 42776–42786 (2023). 38024766 10.1021/acsomega.3c05586 PMC10652833

[21] Noureldeen, A. F. H., Qusti, S. Y. & Al-seeni, M. N. Serum leptin, adiponectin, resistin, visfatin and inflammatory cytokines in normal weight and obese women with normal pregnancy and with preeclampsia. *Life Sci. J.* **11**, 17–23 (2014).

[22] Noureldeen, A. F. H., Qusti, S. Y., Al-seeni, M. N. & Bagais, M. N. Maternal leptin, adiponectin, resistin, visfatin and tumor necrosis factor-alpha in normal and gestational diabetes. *Indian J. Clin. Biochem.* **29**, 462–470 (2014). 25298627 10.1007/s12291-013-0394-0 PMC4175703

[23] Gashlan, H. M., Noureldeen, A. F. H., Elsherif, H. A. & Tareq, O. Vitamin D and insulin resistance in gestational diabetes mellitus. *J. Diabetes Endocrinol.* **8**, 17–25 (2017).

[24] Noureldeen, A. F. H., AlGhamdi, M. A. & Alsolami, Y. S. Z. Maternal status of trace elements in normal pregnancy and in gestational diabetes mellitus. *Int. J. Pharm. Phytopharm. Res.* **8**, 1–9 (2018).

[25] American diabetes association. 2. Classification and diagnosis of diabetes: Standards of medical care in diabetes-2019. Diabetes Care. *42*, S13-S28. (2019). 10.2337/dc19-S002 30559228

[26] a), A. et al. Gestational diabetes mellitus: Major risk factors and pregnancy-related outcomes: A cohort study. Int. J. Reprod. Biomed *19*, 827 – 826. b) D.R. Matthews, J.P. Hosker, A.S. Rudenski, B.A. Naylor, D.F. Treacher, R.C. Turner, Homeostasis model assessment: insulin resistance and β-cell function from fasting plasma glucose and insulin concentrations in man, *diabetologia*, 1985, *28*, 412–419. (2021). 10.1007/BF00280883 3899825

[27] Atakul, N., Atamer, Y., Selek, Ş, Kılıç, B. S. & Unal, F. Novel metabolic marker Afamin: A predictive factor for large-for-gestational-age (LGA) fetus estimation in pregnancies with gestational diabetes mellitus?. *J. Gynecol. Obstet. Hum. Reprod.* **50**, 102201–102206 (2021). 34365029 10.1016/j.jogoh.2021.102201

[28] Yuan, Y. et al. Serum Afamin levels in predicting gestational diabetes mellitus and preeclampsia: A systematic review and meta-analysis. *Front. Endocrinol. (Lausanne)* **14**, 1157114–1157121 (2023). 37033215 10.3389/fendo.2023.1157114 PMC10073667

[29] Köninger, A. et al. Afamin: An early predictor of preeclampsia. *Arch. Gynecol. Obstet.* **298**, 1009–1016 (2018). 30220025 10.1007/s00404-018-4897-z PMC6182689

[30] Gülücü, S., Çelik, S. & Unver, G. Evaluation of first-and third-trimester Afamin levels in preeclampsia. *Rev. Assoc. Med. Bras.* **69**, 430–433 (2023). 36921197 10.1590/1806-9282.20221115 PMC10004282

[31] Tramontana, A., Dieplinger, B., Stangl, G., Hafner, E. & Dieplinger, H. First trimester serum Afamin concentrations are associated with the development of pre-eclampsia and gestational diabetes mellitus in pregnant women. *Clin. Chim. Acta* **476**, 160–166 (2018). 29191735 10.1016/j.cca.2017.11.031

[32] Eroğlu, H., Örgül, G., Tonyalı, N. V., Biriken, D. & Polat, N. The role of Afamin and other trace elements in the prediction of GDM: A tertiary center experience. *Biol. Trace Elem. Res.* **199**, 4418–4422 (2021). 33442846 10.1007/s12011-020-02559-0

[33] Cheng, X., Cui, H., Xu, X. & Jiang, N. Correlation between plasma Afamin and gestational diabetes mellitus during pregnancy. *Ginekol. Pol.*10.5603/gpl.103660 (2025). 40485265 10.5603/gpl.103660

[34] Prabhu, K., Pangaluri, R., Murugesan, A., Mohanakrishnan, V. V. & Sivakumar, R. The role of Afamin in gestational diabetes mellitus: From placental dysfunction to prediction. *J. South. Asian Fed. Obstet. Gynaecol.* **17**, 386–394 (2025).

[35] Köninger, A., Mathan, A., Mach, P. & Frank, M. Is Afamin a novel biomarker for gestational diabetes mellitus? A pilot study. *Reprod. Biol. Endocrinol.* **16** (1), 30–40 (2018). 29587878 10.1186/s12958-018-0338-x PMC5870691

[36] Biondi, C., Pavan, B., Lunghi, L., Fiorini, S. & Vesce, F. The role and modulation of the oxidative balance in pregnancy. *Curr. Pharm. Des.* **11**, 2075–2089 (2005). 15974960 10.2174/1381612054065747

[37] Mannaerts, D., Faes, E., Cos, P., Briede, J. J. & Gyselaers, W. Oxidative stress in healthy pregnancy and preeclampsia is linked to chronic inflammation, iron status and vascular function. *PLoS One* **13**, e0202919 (2018). 30204759 10.1371/journal.pone.0202919 PMC6133366

[38] Dieplinger, B. et al. Analytical characterization and clinical evaluation of an enzyme-linked immunosorbent assay for measurement of Afamin in human plasma. *Clin. Chim. Acta* **425**, 236–241 (2013). 23981841 10.1016/j.cca.2013.08.016 PMC3819992

[39] Li, Q., Li, C., Jin, J., Shen, Y. & Wang, M. Clinical significance of neuregulin 4, afamin, and SERPINB1 in gestational diabetes mellitus and their relationship with insulin resistance. *Evid Based Complement. Altern. Med*, **2022**(1), 2829662–2829669 . 10.1155/2022/2829662 PMC9441345 36072413

[40] Alakbarova, L., Kale, İ & Muhcu, M. Investigation of serum Afamin concentration in pregnant women diagnosed with late fetal growth restriction or small for gestational age fetus. *J. Matern. Fetal Neonatal Med.* **36**, 2240468–2240473 (2023). 37518071 10.1080/14767058.2023.2240468

[41] Bendary, A. & Marei, Y. High serum levels of Afamin and tumor necrosis factor-α during the first trimester might be used as early predictors for gestational diabetes mellitus in euglycemic pregnant women. *Evid. Based Women’s Health J.* **12**, 378–386 (2022).

[42] Hussain, T. et al. Modulatory mechanism of polyphenols and Nrf2 signaling pathway in LPS challenged pregnancy disorders, *Oxid. Med. Cell. Longev. 2017*, 8254289. (2017). 10.1155/2017/8254289 PMC5613688 29138679

[43] Dogan, Y., Arslan, O., Oztas, B. & Kurtali, A. Comparison of Afamin values in umbilical cord blood after delivery in pregnancies with and without gestational diabetes mellitus. *Fetal Pediatr. Pathol.* **43**, 83–93 (2024). 38189115 10.1080/15513815.2023.2300981

[44] Wang, X. et al. The clinical values of Afamin, triglyceride and PLR in predicting risk of gestational diabetes during early pregnancy. *Front. Endocrinol. (Lausanne)* **12**, 723650 (2021). 34803906 10.3389/fendo.2021.723650 PMC8597949

[45] Wu, P. et al. Maternal overweight and obesity modify the association of serum fibroblast growth factor 21 levels with gestational diabetes mellitus: A nested case-control study. *Diabetes. Metab. Res. Rev.* **40**, e3717 (2024). 37649397 10.1002/dmrr.3717

[46] Wang, Z. et al. FGF21 serum levels in the early second trimester are positively correlated with the risk of subsequent gestational diabetes mellitus: A propensity-matched nested case-control study. *Front. Endocrinol. (Lausanne)* **12**, 630287 (2021). 33995273 10.3389/fendo.2021.630287 PMC8113961

[47] Jia, J. et al. Circulating levels of fibroblast growth factor 21 in gestational diabetes mellitus: a meta-analysis. *Endocr. J.* **68**, 345–352 (2021). 33162410 10.1507/endocrj.EJ20-0481

[48] Mosavat, M., Omar, S. Z. & Sthanshewar, P. Serum FGF-21 and FGF-23 in association with gestational diabetes: a longitudinal case-control study, *Horm. Mol. Biol. Clin. Investig. 41*. (2020). 10.1515/hmbci-2019-0060 32167928

[49] Jia, X., Bai, L., Ma, N. & Lu, Q. The relationship between serum adipokine fibroblast growth factor-21 and gestational diabetes mellitus. *J. Diabetes. Investig.* **13**, 2047–2053 (2022). 35871469 10.1111/jdi.13889 PMC9720227

[50] Li, S. M. et al. Fibroblast growth factor 21 expressions in white blood cells and sera of patients with gestational diabetes mellitus during gestation and postpartum. *Endocrine* **48**, 519–527 (2015). 24895044 10.1007/s12020-014-0309-8

[51] Megia, A. et al. Cord blood FGF21 in gestational diabetes and its relationship with postnatal growth. *Acta. Diabetol.* **52**, 693–700 (2015). 25604041 10.1007/s00592-014-0705-9

[52] Wang, D., Zhu, W., Li, J., An, C. & Wang, Z. Serum concentrations of fibroblast growth factors 19 and 21 in women with gestational diabetes mellitus: Association with insulin resistance, adiponectin, and polycystic ovary syndrome history. *PLoS One* **8**, e81190–e81197 (2013). 24260557 10.1371/journal.pone.0081190 PMC3834317

[53] Stein, S. et al. Serum fibroblast growth factor 21 levels in gestational diabetes mellitus in relation to insulin resistance and dyslipidemia. *Metabolism* **59**, 33–37 (2010). 19699495 10.1016/j.metabol.2009.07.003

[54] Xu, L. et al. Elevated plasma SPARC levels are associated with insulin resistance, dyslipidemia, and inflammation in gestational diabetes mellitus. *PLoS One* **8**, e81615-81622 (2013). 24349098 10.1371/journal.pone.0081615 PMC3857203

[55] Gawlik, K., Milewicz, T., Pawlica-Gosiewska, D., Trznadel-Morawska, I. & Solnica, B. Fibroblast growth factor 21 in gestational diabetes mellitus and type 2 diabetes mellitus, *J. Diabetes Res. 2023*, 4024877. (2023). 10.1155/2023/4024877 PMC10590262 37869250

[56] Woo, Y. C. et al. Serum fibroblast growth factor 21 is a superior biomarker to other adipokines in predicting incident diabetes. *Clin. Endocrinol. (Oxf.)* **86**, 37–43 (2017). 27611701 10.1111/cen.13229

[57] Nitert, M. D. et al. Increased placental expression of fibroblast growth factor 21 in gestational diabetes mellitus. *J. Clin. Endocrinol. Metab.* **99**, E591–E598 (2014). 24432989 10.1210/jc.2013-2581

[58] Xu, C., Han, Z., Li, P. & Li, X. Fibroblast growth factor 21 is a potential diagnostic factor for patients with gestational diabetes mellitus. *Exp. Ther. Med.* **16**, 1397–1402 (2018). 30116389 10.3892/etm.2018.6291 PMC6090206

[59] Kharitonenkov, A. et al. FGF-21 as a novel metabolic regulator. *J. Clin. Invest.* **115**, 1627–1635 (2005). 15902306 10.1172/JCI23606 PMC1088017

[60] Kharitonenkov, A., Wroblewski, V. J., Koester, A. & Chen, Y. F. The metabolic state of diabetic monkeys is regulated by fibroblast growth factor-21. *Endocrinology* **148**, 774–781 (2007). 17068132 10.1210/en.2006-1168

[61] W. JM, *Fibroblast growth factor 21 (FGF21) resistance in adipose tissue is associated with the pathogenesis of gestational diabetes mellitus in humans* (Dissertation), Guangdong Pharmaceutical University, (2019).

[62] Fisher, F. M. et al. Maratos-Flier Obesity is a fibroblast growth factor 21 (FGF21)-resistant state. *Diabetes* **59**, 2781–2789 (2010). 20682689 10.2337/db10-0193 PMC2963536

[63] Pan, Y. et al. Pancreatic fibroblast growth factor 21 protects against type 2 diabetes in mice by promoting insulin expression and secretion in a PI3K/Akt signaling-dependent manner. *J. Cell. Mol. Med.* **23**, 1059–1071 (2019). 30461198 10.1111/jcmm.14007 PMC6349243

[64] Zhang, X. et al. Serum FGF21 levels are increased in obesity and are independently associated with the metabolic syndrome in humans. *Diabetes* **57**, 1246–1253 (2008). 18252893 10.2337/db07-1476

[65] Traisrisilp, K., Apaijai, N., Waisayanand, N. & Chattipakorn, S. Serum fibroblast growth factor 21: Lack of association with gestational diabetes and pregnancy outcomes. *World J. Obstet. Gynecol.* **13**, 100776–100784 (2024).

[66] Bonakdaran, S., Khorasani, Z. M. & Jafarzadeh, F. Increased serum level of FGF21 in gestational diabetes mellitus. *Acta Endocrinol. (Bucharest)*. **13**, 278–281 (2017). 10.4183/aeb.2017.278 PMC6516566 31149188

