# Supplementary material for: Efficiency of afamin and fibroblast growth factor 21 for early prediction of gestational diabetes mellitus
Source: Sci Rep. 2026 May 11;16:14716. doi: 10.1038/s41598-026-51453-7 (PMC13161233; doi:10.1038/s41598-026-51453-7)

**Supplementary file**

**Efficiency of Afamin and FGF21 for Early Prediction of Gestational Diabetes Mellitus**

Abdelrahman O. Sabri^1^, Mostafa M. Elhady^1^, Eman H.A. Hemida^2^, Radwa M.M. Zaki^3^ and Amani F.H. Noureldeen^1,*^

^1^ Department of Biochemistry, Faculty of Science, Ain Shams University, Cairo, Egypt.

^2^ Obstetrics and Gynecology Hospital, Ain Shams University, Cairo, Egypt.

^3^ Obstetrics and Gynecology Department, Faculty of Medicine, Ain Shams University, Cairo, Egypt.

* Corresponding author e-mail address: [amaninoureldeen@yahoo.com](mailto:amaninoureldeen@yahoo.com)

**Table S1:** Correlation of afamin with biochemical parameters and clinical characteristics at 1^st^ trimester.

| **Groups**  **Parameters** | **Normal** | | **GDM** | |
| --- | --- | --- | --- | --- |
|  | ***R*** | ***P*** | ***r*** | ***P*** |
| BMI (Kg/m^2^) | 0.440 | 0.077 (NS) | 0.508 | 0.008 |
| FBG (mg/dl) | 0.475 | 0.054 (NS) | 0.620 | 0.001 |
| FI (mIU/ml) | 0.420 | 0.093 (NS) | 0.533 | 0.005 |
| HbA1C (%) | 0.464 | 0.06 1(NS) | 0.425 | 0.03 |
| HOMA-IR | 0.428 | 0.087 (NS) | 0.551 | 0.004 |
| ALT (IU/L) | 0.336 | 0.187 (NS ) | 0.218 | 0.285 (NS) |
| AST (IU/L) | 0.281 | 0.275 (NS) | 0.90 | 0.662 (NS) |
| Albumin (g/dl) | 0.086 | 0.742 (NS) | -0.285 | 0.158 (NS) |
| Urea (mg/dl) | 0.086 | 0.742 (NS) | 0.207 | 0.311 (NS) |
| Creatinine (mg/dl) | -0.285 | 0.268 (NS) | 0.085 | 0.679 (NS) |
| Uric acid (mg/dl) | 0.396 | 0.115 (NS) | 0.322 | 0.109 (NS) |
| Birth Weight (Kg) | 0.644 | 0.085 (NS) | 0.531 | 0.042 |

BMI: Body mass index, FBG: Fasting blood glucose, FI: Fasting insulin, HBA1C: Hemoglobin A1C, HOMA-IR: Homeostatic Model Assessment of Insulin Resistance, ALT: Aspartate Aminotransferase, AST: Aspartate Aminotransferase.

*P* value ≤ 0.05 is significant. *P* value > 0.05 is non-significant, NS: non-significant.

*r*: Pearson correlation coefficient.

**Table S2:** Correlation of afamin with biochemical parameters and clinical characteristics at 2^nd^ trimester.

| **Groups Parameters** | **Normal** | | **GDM** | |
| --- | --- | --- | --- | --- |
|  | ***R*** | ***P*** | ***r*** | ***P*** |
| BMI (Kg/m^2^) | 0.397 | 0.114 (NS) | 0.212 | 0.299 (NS) |
| FBG (mg/dl) | 0.380 | 0.133 (NS) | 0.393 | 0.047 |
| FI (mIU/ml) | 0.489 | 0.046 | 0.321 | 0.110 (NS) |
| HbA1C (%) | 0.469 | 0.057 (NS) | 0.247 | 0.224 (NS) |
| HOMA-IR | 0.399 | 0.113 (NS) | 0.354 | 0.076 (NS) |
| ALT (IU/L) | 0.361 | 0.155 (NS) | 0.280 | 0.167 (NS) |
| AST (IU/L) | 0.422 | 0.092 (NS) | 0.260 | 0.200 (NS) |
| Albumin (g/dl) | 0.144 | 0.580 (NS) | 0.167 | 0.389 (NS) |
| Urea (mg/dl) | 0.195 | 0.453 (NS) | -0.300 | 0.136 (NS) |
| Creatinine (mg/dl) | 0.247 | 0.340 (NS) | 0.368 | 0.065 (NS) |
| Uric acid (mg/dl) | 0.230 | 0.375 (NS) | 0.011 | 0.958 (NS) |
| Birth Weight (Kg) | 0.231 | 0.094 (NS) | -0.127 | 0.536 (NS) |

**Table S3:** Correlation of FGF21 with biochemical parameters and clinical characteristics at 1^st^ trimester.

| **Groups**  **Parameters** | **Normal** | | **GDM** | |
| --- | --- | --- | --- | --- |
|  | ***R*** | ***P*** | ***r*** | ***P*** |
| BMI (Kg/m^2^) | 0.171 | 0.511 (NS) | 0.463 | 0.082 (NS) |
| FBG (mg/dl) | 0.422 | 0.091 (NS) | 0.361 | 0.07 (NS) |
| FI (mIU/ml) | 0.132 | 0.613 (NS) | 0.282 | 0.163 (NS) |
| HbA1C (%) | 0.443 | 0.075(NS) | 0.627 | 0.048 |
| HOMA-IR | 0.312 | 0.223 (NS) | 0.325 | 0.105 (NS) |
| ALT (IU/L) | 0.021 | 0.937 (NS) | 0.099 | 0.631 (NS) |
| AST (IU/L) | 0.052 | 0.844 (NS) | -0.048 | 0.814 (NS) |
| Albumin (g/dl) | 0.045 | 0.864 (NS) | -0.111 | 0.588 (NS) |
| Urea (mg/dl) | 0.136 | 0.603 (NS) | 0.024 | 0.907 (NS) |
| Creatinine (mg/dl) | -0.135 | 0.605 (NS) | -0.221 | 0.278 (NS) |
| Uric acid (mg/dl) | 0.100 | 0.702 (NS) | 0.061 | 0.766 (NS) |
| Birth Weight (Kg) | 0.272 | 0.515 (NS) | 0.471 | 0.076 (NS) |

**Table S4:** Correlation of FGF21 with biochemical parameters and clinical characteristics at 2^nd^ trimester.

| **Groups**  **Parameters** | **Normal** | | **GDM** | |
| --- | --- | --- | --- | --- |
|  | ***R*** | ***P*** | ***r*** | ***P*** |
| BMI (Kg/m^2^) | 0.397 | 0.114 (NS) | 0.276 | 0.283 (NS) |
| FBG (mg/dl) | 0.380 | 0.133 (NS) | 0.257 | 0.3209 (NS) |
| FI (mIU/ml) | 0.489 | 0.046 | 0.149 | 0.568 (NS) |
| HbA1C (%) | 0.469 | 0.057 (NS) | 0.323 | 0.206 (NS) |
| HOMA-IR | 0.399 | 0.113 (NS) | 0.439 | 0.078 (NS) |
| ALT (IU/L) | 0.361 | 0.155 (NS) | 0.440 | 0.077 (NS) |
| AST (IU/L) | 0.422 | 0.092 (NS) | 0.460 | 0.063 (NS) |
| Albumin (g/dl) | 0.144 | 0.580 (NS) | 0.084 | 0.749 (NS) |
| Urea (mg/dl) | 0.195 | 0.453 (NS) | 0.098 | 0.709 (NS) |
| Creatinine (mg/dl) | 0.247 | 0.340 (NS) | 0.088 | 0.737 (NS) |
| Uric acid (mg/dl) | 0.230 | 0.375 (NS) | 0.157 | 0.547 (NS) |
| Birth Weight (Kg) | 0.231 | 0.094 (NS) | 0.316 | 0.446 (NS) |

**Table S5:** Diagnostic values of afamin, FGF21 and merged afamin with FGF21 at 1^st^ and 2^nd^ trimesters in normal pregnancy and GDM groups.

| **Groups** | **GDM vs. Control pregnancy** | | | | | |
| --- | --- | --- | --- | --- | --- | --- |
|  | **Afamin** | | **FGF21** | | **Merged afamin and FGF21** | |
|  | **1^st^ trimester** | **2^nd^ trimester** | **1^st^ trimester** | **2^nd^ trimester** | **1^st^ trimester** | **2^nd^ trimester** |
| AUC | 0.826 | 0.575 | 0.877 | 0.753 | 0.900 | 0.751 |
| *P* value | <0.001 | 0.412 | <0.001 | 0.005 | <0.001 | 0.006 |
| Cut off value | 60.5 | 76.5 | 116.5 | 89 |  |  |
| Sensitivity % | 96.15 | 42.30 | 65.38 | 92.30 | 91.66 | 77.41 |
| Specificity % | 58.82 | 70.58 | 88.23 | 58.82 | 78.94 | 83.3 |
| Positive group (%) | 60.47 | 60.47 | 60.47 | 60.47 | 26 | 26 |
| Negative group (%) | 39.53 | 39.53 | 39.53 | 39.53 | 17 | 17 |
| Standard error | 0.66 | 0.089 | 0.053 | 0.079 | 0.049 | 0.079 |
| 95% Confidence interval | 0.697-0.954 | 0.401-0.749 | 0.773-0.981 | 0.685-0.948 | 0.805-0.996 | 0.596-0.906 |
| PPV % | 78.1 | 68.75 | 89.47 | 77.41 | 84.61 | 92.30 |
| NPV % | 81.4 | 55.55 | 62.5 | 83.33 | 88.23 | 58.82 |
| Accuracy % | 81.39 | 53.48 | 74.41 | 79.06 | 86.04 | 79.06 |

AUC: area under the curve, PPV: positive predictive value, NPV: negative predictive value.

**Ethics approval and consent to participate**


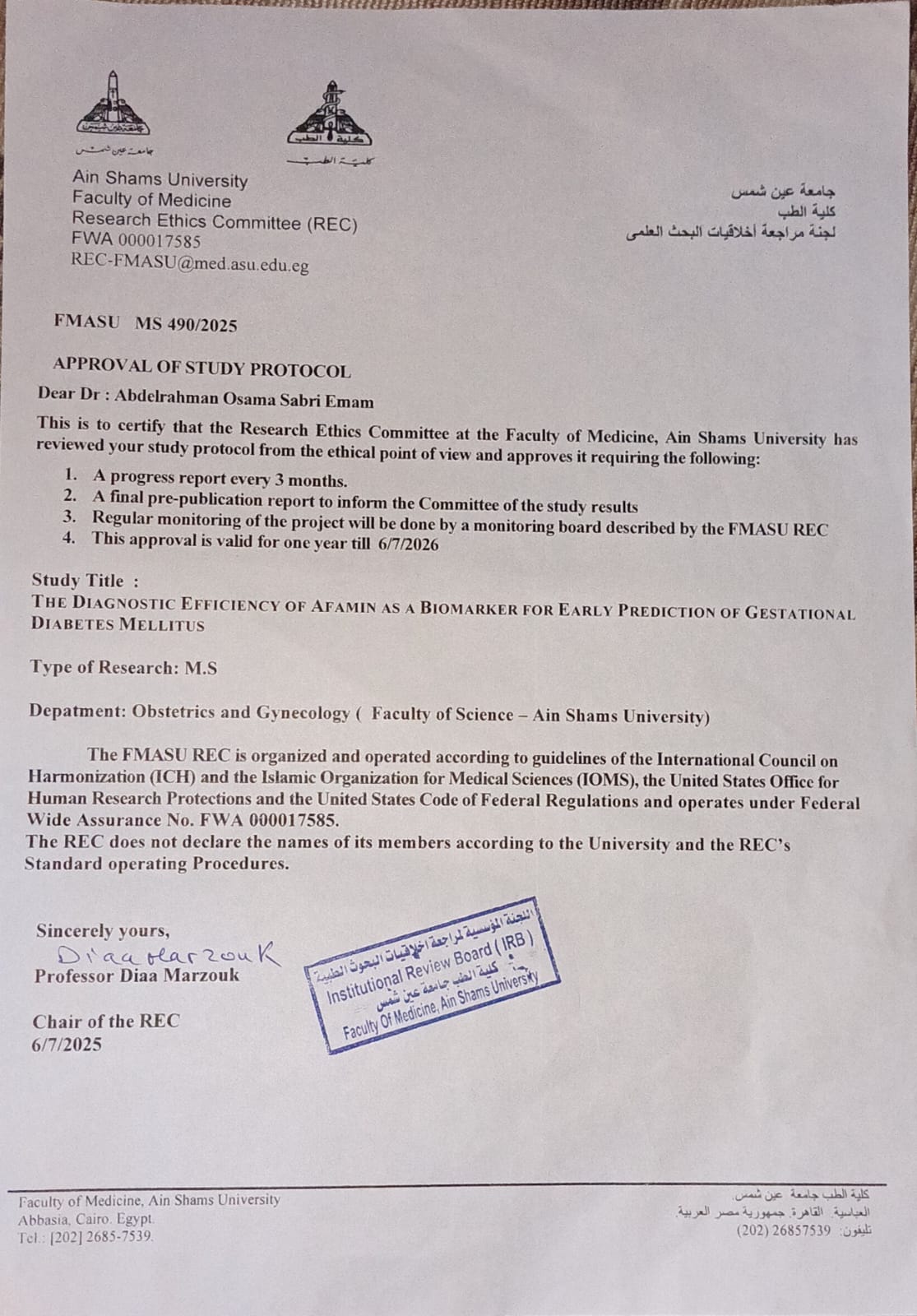

Supplement: Supplementary file 2 — Supplementary Material 2 [file 41598_2026_51453_MOESM2_ESM.docx]
